# Co-occurring Trajectories of Direct Aggression and Prosocial Behaviors in Childhood: Longitudinal Associations With Peer Acceptance

**DOI:** 10.3389/fpsyg.2020.581192

**Published:** 2020-11-30

**Authors:** Idean Ettekal, Minoo Mohammadi

**Affiliations:** Department of Educational Psychology, College of Education and Human Development, Texas A&M University, College Station, TX, United States

**Keywords:** aggression, prosocial behaviors, trajectories, resource control, peer acceptance, social preference, childhood

## Abstract

This study examined the longitudinal associations among children’s direct (physical and verbal) aggression, prosocial behaviors, and peer group acceptance in middle childhood (Grades 1 to 4). Children’s co-occurring aggressive and prosocial behaviors were assessed in order to identify distinct trajectory subgroups. Subsequently, variations in the development (i.e., continuity and changes) in peer acceptance were examined for each of the identified subgroups. The sample consisted of 784 children who were ethnically and socioeconomically diverse (47% girls, 37.4% Latino or Hispanic, 34.1% European American, and 23.2% African American; about 65% low SES) who were followed longitudinally from Grades 1 to 4 (*M*_*age*_ = 6.57 years old in Grade 1). Results revealed several distinct trajectory subgroups, including children who were primarily aggressive or prosocial, as well as children who exhibited co-occurring aggression and prosocial behaviors. Comparing these subgroups, the use of co-occurring prosocial behaviors appeared to have some protective effect on aggressive children’s peer acceptance. However, aggression was nonetheless associated with lower peer acceptance. The findings provide insights pertaining to the heterogeneity among aggressive children, the protective effects of prosocial behaviors on peer acceptance, and the differential effects of moderate versus high aggression.

## Introduction

One of the most salient developmental tasks that children encounter during their grade school years is to form relationships with peers and classmates. A substantial body of research has focused on the construct of peer acceptance, which reflects a child’s degree of likability within their peer group. Although peer acceptance has been found to forecast positive adjustment outcomes across childhood and adolescence, there is considerable variability among children with respect to how well-liked they are by peers (Parker and Asher, 1987; Newcomb et al., 1993; Ladd, 2005). In an effort to elucidate what factors impact peer acceptance, researchers have examined the role of children’s behavioral styles, and more specifically, aggression. One of the most consistent findings to emerge is that direct forms of aggression, such as physical and verbal aggression, are inversely associated with peer acceptance and positively associated with peer rejection (Card et al., 2008). However, these associations tend to be moderate in strength, implying that some physically aggressive children are able to maintain higher levels of peer acceptance. Consequently, there has been a growing interest in examining the heterogeneity and individual differences among aggressive children. Building on this work, in the current study, we evaluate the premise that prosocial behaviors may buffer (or reduce) the negative effects of direct aggression on children’s peer acceptance. More specifically, the primary aims of the present study were twofold: (1) to examine the co-occurring (or joint) developmental trajectories of direct aggression and prosocial behaviors from middle to late childhood (Grades 1 to 4), and (2) to assess how variations (i.e., individual differences) in these behavioral trajectories were associated with continuity and changes in children’s peer acceptance.

### Development of Direct (Physical and Verbal) Aggression

Several general conclusions can be drawn from studies which have investigated the development and continuity of direct forms of aggression in childhood. Earlier research, which primarily utilized variable centered research designs, provided consistent evidence that there is rank-order stability in aggression over time (Olweus, 1979; Ettekal and Ladd, 2009). Notwithstanding this rank-order stability, direct forms of aggression tend to exhibit a normative decline across childhood and adolescence as most children develop more advanced emotion and self-regulation, social-cognitions, communication, verbal skills, and conflict resolution strategies (Loeber and Hay, 1997; Tremblay, 2010; Ettekal and Ladd, 2017).

Despite these normative declines, studies which have applied person-centered designs have consistently found that children exhibit individual differences and heterogeneity in their developmental trajectories of aggression. Numerous longitudinal studies have investigated the developmental trajectories of aggressive behaviors across a wide range of developmental periods, demographic groups (e.g., gender, race, ethnicity, nationality, and socioeconomic status), and methodological factors (e.g., different informant types, measures, and forms of aggression). Across these studies, there have typically been between two to five distinct trajectory subgroups, with most studies identifying three: children with low, moderate, or high (chronic) levels of aggression (Broidy et al., 2003; Barker et al., 2006; Campbell et al., 2006; Kokko et al., 2006; Vaillancourt et al., 2007; Martino et al., 2008; Ehrenreich et al., 2014; Nantel-Vivier et al., 2014; Ettekal and Ladd, 2015a; Girard et al., 2019). These trajectories appear to be either relatively stable over time or declining, and the majority of children are classified in the low or moderate groups.

### Development of Prosocial Behaviors

Prosocial behaviors refer to voluntary actions that benefit other people (Eisenberg et al., 2015). Although there is consensus that prosocial behaviors emerge in early childhood, findings have been mixed with respect to the normative developmental progression of prosocial behaviors in childhood and adolescence. On the one hand, as children’s social-cognitive, emotional and moral development progresses (e.g., empathy, perspective-taking skills), there may be corresponding increases in certain forms of prosocial behaviors (Eisenberg and Fabes, 1998). On the other hand, children may become more selective in their use of prosocial behaviors, coinciding with either a normative decline or stability across time (Hay and Cook, 2007; Eisenberg et al., 2015).

In addition to examining normative trends, several longitudinal studies have used person-centered methods to examine heterogeneous developmental trajectories of prosocial behavior. Although these studies have varied with respect to the developmental periods investigated and methodological factors (e.g., measures of prosocial behavior and informant types), the findings indicate between two to four distinct trajectory subgroups across childhood and adolescence (Côté et al., 2002; Kokko et al., 2006; Nantel-Vivier et al., 2009, 2014; Flynn et al., 2015; Shi et al., 2020). Moreover, three subgroups, characterized by either low, moderate, or high levels of prosocial behavior have been identified most consistently, with most children exhibiting moderate rates. Notably, there have been some variations in the continuity or discontinuity (i.e., slopes) of prosocial behaviors among different subgroups. For instance, one study focusing on early and middle childhood (ages 2–11 years old) found that all three trajectory classes (low, moderate, and high) exhibited increases in prosocial behavior (Nantel-Vivier et al., 2014). In contrast, across studies focusing on middle and late childhood and adolescence, most trajectory classes were either stable or slightly declining across time.

### Co-occurring (Joint) Development of Aggressive and Prosocial Behaviors

With a few exceptions, most studies on the developmental trajectories of aggression and prosocial behaviors have investigated these constructs independently. Part of the rationale for this trend is based on conceptual frameworks which consider aggressive and prosocial behaviors as reflecting opposing ends of a continuum (Wispe, 1972; Obsuth et al., 2015). Indeed, conceptualizations of aggression, which posit that it stems from social-skills deficits and social cognitive biases (Dodge et al., 2003), imply that aggressive children are unable or unwilling to engage in prosocial behaviors. Although it is plausible that many aggressive children may be lacking the social skills or competencies which foster the use of prosocial behaviors, investigators have also proposed alternative perspectives which posit that children who engage in aggression represent a heterogeneous population and exhibit substantial variation in their co-occurring use of prosocial behaviors (Rodkin et al., 2000; Kokko et al., 2006).

To evaluate these alternative perspectives, there have been a few empirical studies by researchers who have applied person-centered longitudinal designs to examine the joint developmental trajectories of aggression and prosocial behaviors. Taken together, several conclusions can be drawn from these studies. First, most children tend to exhibit a combination of low aggression and moderate prosocial behaviors. Second, when children are highly aggressive, they tend to exhibit low or moderate levels of prosocial behavior. For instance, Kokko et al. (2006) examined the co-occurring development of aggression and prosocial behavior in boys from 6 to 12 years old. They found that the majority (79.3%) of highly aggressive boys exhibited low levels of prosocial behavior; however, there was also a subgroup (20.7%) of highly aggressive boys with moderate prosocial behaviors (notably, they did not identify a high prosocial trajectory class). Nantel-Vivier et al. (2014) used a similar joint trajectory modeling approach to examine co-occurring aggressive and prosocial behaviors in children from ages 2 to 11 years. They reported that only 2% of children had high aggression and high prosocial trajectories, and among children with high aggression trajectories, the vast majority had either low or moderate prosocial trajectories. Thus, these findings are consistent with the premise that aggression and prosocial behaviors tend to be negatively correlated.

A third conclusion drawn from this line of research is that when children are engaging in both aggression and prosocial behaviors, they tend to be exhibited at moderate levels. For instance, Pouwels et al. (2018) examined a sample of children from Grades 3 to 8, and one of the classes they identified was characterized by moderate-increasing aggression and moderate-low decreasing prosocial behaviors (10.6%). In contrast, Jambon et al. (2019) examined a sample of preschool children (ages 3–6 years old), and two of the classes they identified were characterized by moderate-declining aggression with moderate-increasing prosocial behavior (19.6%) or moderate-increasing aggression with moderate prosocial behaviors (19.3%). As can be gleaned from these findings, there also appears to be some variability across studies with respect to the slopes (e.g., increasing, decreasing, stable) of different co-occurring trajectory classes, particularly among children with more moderate levels of aggression and prosocial behaviors. In conclusion, these findings reflect individual differences (i.e., heterogeneity) among aggressive children, such that some exhibit co-occurring (albeit moderate) prosocial behaviors in contrast to others who are primarily aggressive (i.e., with low prosocial behaviors).

### Direct Aggression, Prosocial Behaviors, and Peer Acceptance

Research on aggression and prosocial behavior indicates that children’s behavioral styles have a substantial impact on different facets of their peer relationships, including their peer acceptance. With respect to main effects, there is substantial evidence that children’s prosocial behaviors are positively associated with peer acceptance (Pakaslahti et al., 2002; Eisenberg et al., 2006; Kuppens et al., 2009; Carlo et al., 2012; Caprara et al., 2014; Wang et al., 2019). When children engage in cooperative behaviors (e.g., sharing, helping, instrumental, social, and emotional support), they are likely to foster a harmonious social climate that encourages their peers to reciprocate these behaviors. These positive interaction styles make children desirable friends and playmates, and promote their reputation as being highly likable. In contrast, direct aggression is likely to lead to lower peer acceptance and greater peer rejection (Card et al., 2008; Carlo et al., 2012; Ettekal and Ladd, 2015a, b; Wang et al., 2019). When children engage in threatening and confrontational behaviors, they are likely to foster a hostile social climate. These interaction styles are likely to make children aversive and discourage their peers from interacting and playing with them.

Although the independent associations among aggressive or prosocial behaviors and peer acceptance have been consistently substantiated in studies examining main effects, there is also a growing body of evidence which suggests that under certain conditions, direct aggression may be adaptive (i.e., positively associated) with respect to children’s peer acceptance. Based on extant evidence and theory, we evaluate two potential propositions in the current study. The first proposition pertains to how co-occurring prosocial behaviors may buffer the effects of direct aggression, and the second proposition pertains to the potential adaptive role of moderate (as opposed to high) levels of aggression.

With respect to the first proposition, several investigators have evaluated the extent to which prosocial behavior has a protective effect in which it reduces the negative impact of aggression; however, the empirical evidence has been mixed and inconclusive. For instance, using a cross-sectional design, Bierman et al. (1993) reported that aggressive and aggressive-rejected boys had similar levels of prosocial behavior; thus, it did not appear that prosocial behaviors differentiated aggressive children with varying levels of peer rejection. Expanding on these findings, Kokko et al. (2006) used a longitudinal person-centered design to examine boys’ co-occurring aggression and prosocial trajectories; however, they did not find support for the protective effects of prosocial behavior on school dropout or violence (notably, this study did not specifically evaluate peer acceptance as an outcome variable).

Several studies have also applied resource control theory to examine differential outcomes among children identified as bistrategic controllers in contrast to coercive (aggressive) controllers. Studies using this approach typically classify (categorize) children into one of five distinct resource control subgroups (e.g., Hawley et al., 2002; Hawley, 2003a). Subgroups are created by first measuring children’s coercive and prosocial strategies and then using cut-off scores (e.g., using the top third percentile) to categorize children. More specifically, prosocial controllers score high on prosocial strategies (but low on coercive strategies), coercive controllers score high on coercive strategies only, and bistrategic controllers score in the top third on both strategies. In addition to these three groups, non-controllers score the lowest on both strategies (e.g., bottom third), and typical controllers score in the average range (e.g., middle third). Using this categorization scheme, bistrategic controllers have been hypothesized to maintain power (‘getting ahead’) and social harmony (‘getting along’; see Hogan, 1982). That is, although they are aggressive, they are also viewed as being socially competent and balancing their use of aggression with prosocial behaviors to reduce potential social sanctions and retributions, thus ameliorating the negative effects of aggression (Roseth et al., 2011). Consistent with this viewpoint, several investigators have reported that bistrategic controllers had higher social preference than typical and coercive controllers, and similar levels as prosocial controllers (Hawley, 2003b; Wurster and Xie, 2014). Contrary to these findings, other investigators have reported that bistrategic controllers may be disliked, and routinely identified by peers as being an enemy, thus exhibiting low peer acceptance or social preference (Reijntjes et al., 2018).

Although the reasoning for these discrepant findings is unclear, one potential explanation may relate to the methodological approach used in these studies in which they rely on arbitrary cut-off scores and sample-specific groupings. Indeed, investigators who have applied person-centered methods which do not rely on cut-off scores (e.g., latent class analysis, latent profile analysis, growth mixture modeling) have yielded somewhat different subgroups and insights. For instance, bistrategic children appear to exhibit moderate (and less severe) levels of aggression than children in the coercive group (Ciarrochi et al., 2019; Jambon et al., 2019; Hartl et al., 2020). Thus, these findings imply that the severity (or levels) of aggression should also be evaluated when (1) identifying children who are both aggressive and prosocial, and (2) examining how co-occurring aggressive and prosocial behaviors are associated with peer acceptance or other child adjustment outcomes. That is, if children are using aggression and prosocial behaviors in planned and instrumental ways to advance their social positions and peer relationships, their aggressive behavioral styles are more likely to be functional and socially acceptable when aggression is used moderately, as opposed to excessively (Ettekal and Ladd, 2015a). In contrast, chronic (and highly frequent) aggression is more likely to reflect an impulsive, emotionally dysregulated, and reactive behavioral style, which aligns more closely with social-cognitive deficit perspectives of aggression (Ettekal and Ladd, 2015a; Olson et al., 2017; Jambon et al., 2019).

### Study Aims and Hypotheses

The current study sought to provide insights into the potential protective effects of prosocial behavior on aggressive children’s peer acceptance, and the specific role of moderate (as opposed to high) levels of direct aggression. Toward this end, the primary aims of the present study were twofold: (1) to examine the co-occurring (or joint) developmental trajectories of direct aggression and prosocial behaviors from middle to late childhood (i.e., Grades 1 to 4), and (2) to assess how variations in these (co-occurring) behavioral trajectories were associated with continuity and changes in children’s peer acceptance.

With respect to the first aim, we first examined the developmental trajectories of aggression and prosocial behaviors separately. Based on extant evidence (previously discussed), we hypothesized identifying at least three relatively stable prosocial and aggression trajectory classes (low, moderate, and high). Subsequently, we examined co-occurring (joint) trajectory classes and expected the possibility of nine (3 × 3) co-occurring classes. However, it was also possible that there would be a very low proportion of children who were both highly aggressive and prosocial; thus this class may not be reliably identified (see Nantel-Vivier et al., 2014).

To assess these joint trajectory classes, we relied on peer reports of direct (physical and verbal) aggression and teacher reports of prosocial behaviors. The reasoning for using these informants was based on multiple considerations. First, this approach minimized potential concerns about shared method variance influencing class identification. Second, peer reports tend to reflect reputational measures of children’s behavioral styles. Thus, once a child is perceived by peers as being aggressive, their use of other (non-aggressive) behaviors may be overshadowed or overlooked. Third, although teachers are highly observant and valid raters of children’s prosocial behaviors (Griese et al., 2016), they may be less aware of aggressive behaviors when enacted by children who are more strategic and skilled at hiding their aggression from authority figures (Hawley, 2003a). Thus, peer-reports may be more reliable than teacher-reports in terms of measuring aggressive exchanges among classmates (Hawley, 2003b). Notwithstanding these potential informant differences, teacher- and peer-reports of aggressive and prosocial behaviors tend to be moderately correlated (Crick, 1996; Greener, 2000).

Our second aim was to chart the development (continuity and changes) in peer acceptance (from Grades 1 to 4) for each of the subgroups identified in Aim 1. Consistent with the proposition that prosocial behaviors have a protective effect, we expected that children who engage in co-occurring aggressive and prosocial behaviors are more likely to exhibit higher levels or increases in peer acceptance as they transition from middle to late childhood, compared to children who were primarily (only) aggressive. Consistent with the proposition that moderate levels of aggression may be adaptive, we hypothesized that (1) moderate aggressors would have higher levels of peer acceptance than high aggressors, and (2) the protective effects of prosocial behavior would be more pronounced in moderately (as opposed to highly) aggressive children.

While examining the associations among co-occurring aggression and prosocial behaviors with peer acceptance, we also controlled for the effects of gender, socioeconomic status, race, and ethnicity. There is substantial evidence that boys tend to be more aggressive than girls (Card et al., 2008), and that boys are overrepresented in joint trajectory classes that exhibit higher aggression and lower prosocial behaviors (Nantel-Vivier et al., 2014). Similar patterns have been reported for children with lower socioeconomic status (Nantel-Vivier et al., 2014). Although the proportion of boys and girls may vary in different trajectory classes, they exhibit relatively similar trajectory profiles (Côté et al., 2002); thus we decided to examine their trajectories in the same model, as opposed to examining boys and girls separately.

## Materials and Methods

### Participants

Participants were 784 children who were part of a multi-ethnic, predominantly low-income sample recruited when they were in the first grade from three different school districts (one urban and two small cities) in Texas as part of a longitudinal study called ‘Project Achieve.’ The average age of participants was 6.57 when they were in Grade 1. About 47.0% of children were girls, 37.4% were Latino/Hispanic, 34.1% were European American, 23.2% were African American, and about 5.4% were Asian, Native American, or Pacific Islander. About 65.0% of participants were low SES as indicated by income-based eligibility for free/reduced lunch, and 42.5% had parents with a high school diploma or less educational attainment. The broader focus of Project Achieve was to examine educational and psychological outcomes in an academically at-risk sample; thus children were eligible to participate if they scored below the median in their school district on a district-administered test of literacy (administered in the spring of Kindergarten or the fall of Grade 1). Additional eligibility criteria included not receiving special education services, speaking English or Spanish as a first language, and not having been previously retained in Grade 1. Due to the sampling design and eligibility criteria, children were recruited from a large number of classrooms and schools, and also became increasingly dispersed over time. Data from this project are publicly available for secondary data analysis at the National Institute of Child Health and Human Development (NICHD) Data and Specimen Hub (DASH^1^).

### Procedure

This study uses four waves of multi-informant data from school districts, teacher-, and peer-reports, collected on an annual basis, toward the end of the school year (from Grade 1 to Grade 4). Participating school districts provided the research staff with information on children’s demographic background (i.e., age, gender, race, ethnicity, and eligibility for free or reduced-price lunch). Teachers completed questionnaires assessing students’ behavioral adjustment (i.e., prosocial behaviors). Peer-report data were used to assess children’s aggression (peer nominations) and peer acceptance (peer ratings). Peer-reports were obtained via individual interviews conducted by the research staff with participating children and their classmates (those with written parental consent). Across classrooms, the median number of children providing ratings and nominations was 12. Peer report data was available for 73.2–79.0% of participants across grade levels. Missing data analyses indicated no significant differences (with respect to children’s gender, race, ethnicity, and socioeconomic adversity) between children who had peer report data and those with missing data. Peer report data was used from classrooms with at least a 40% participation rate, and the mean rate of participation across these classrooms was 65% (range = 40–95%). This cut-off is consistent with the results reported by Marks et al. (2013), who found that peer nominations of overt aggression demonstrate adequate reliability even when participation rates are as low as 40%. Notably, these investigators focused on peer nominations and did not examine reliability estimates for peer ratings (e.g., ratings of peer acceptance). Moreover, McKown et al. (2011) reported that the reliability of peer reports of social preference appears to be highly resistant to random non-participation (e.g., lack of parental consent, school absence) even when participation rates are as low as 40%.

### Measures

#### Direct Aggression

Peer reports of direct (physical and verbal) aggression were obtained via classroom sociometric assessments. Children were asked to provide unlimited nominations of classmates who fit the following description, “Some kids start fights, say mean things, or hit others.” To adjust for the number of nominators (classroom size), the number of nominations received were counted per student and then standardized within the classroom. The procedures used in the current study were consistent with those recommended in the sociometric assessment literature, and peer nominations of aggression have been found to provide valid assessments of children’s aggression (Cillessen and Bukowski, 2000).

#### Prosocial Behavior

Each year, teachers completed the 25-item Strengths and Difficulties Questionnaire (SDQ: Goodman, 1997) using a 3-point Likert-scale (*0 = “not true,” 1 = “somewhat true,” 2 = “certainly true”*). Five items from the Prosocial Behaviors subscale (“considerate of other people’s feelings,” “shares readily with other children,” “helpful if someone is hurt,” “often volunteers to help others,” “kind to younger children”) were summed to form a composite scale score. To convert this scale to the same metric as the aggression measure, these scores were then standardized across the sample. The SDQ has been widely used in educational and psychological research and has demonstrated adequate validity and reliability (Goodman, 1997; Goodman and Goodman, 2009; Stone et al., 2010). Within the current sample, this scale exhibited adequate reliability over time (alphas ranged from 0.84 to 0.86).

#### Peer Acceptance

Peer ratings of peer acceptance were assessed using roster and rating sociometric procedures. First, the interviewer read the names of all the children in the classroom to prompt the child to think about each classmate. Subsequently, the interviewer named each child in the classroom, and asked the child to point to one of five faces ranging from a sad face (1 = don’t like at all) to a happy face (5 = like very much). Each child’s peer acceptance score was derived from taking their average rating across all nominators in their classroom, with higher scores indicating higher levels of peer acceptance. Peer ratings of peer acceptance have been widely used in peer relations research and are valid and reliable indicators of children’s likeability and social status (see Asher et al., 1979; Asher and Dodge, 1986).

#### Socioeconomic Adversity

Based on both school records and parents’ reports, family socioeconomic adversity was calculated as the mean of the standardized scores on five domains: (1) eligibility for free or reduced lunch, (2) single parent status, (3) rental status, (4) occupational level of any adult in the home (coded 1–9; e.g., 9 = farm laborers/menial service workers; 5 = clerical and sales work; 1 = higher executives, proprietors of large businesses), and (5) highest education level of any adult in the home (reverse coded).

### Data Analysis Plan

First, preliminary analyses assessed patterns of missing data, descriptive statistics, and bivariate correlations. Second, growth mixture modeling (GMM) was performed to examine patterns of continuity and change in children’s aggression and prosocial behaviors. More specifically, distinct and heterogeneous trajectory classes were identified from Grades 1 to 4 by first examining aggression and prosocial behaviors independently. After determining the optimal number of trajectory classes for each behavioral domain separately, sequential process growth mixture models were specified to assess the co-occurring development of aggression and prosocial behavior. These models allow for the simultaneous examination of children’s aggression and prosocial behavior trajectories, and estimate the proportion of children who exhibit different combinations of these behavioral styles (e.g., high aggression and high prosocial, or high aggression and low prosocial, etc.). Third, conditional latent growth models were used to examine the development of peer acceptance based on children’s co-occurring aggression and prosocial trajectories. Models were estimated in Mplus (version 8; Muthén and Muthén, 2017). Because of the nested data structure (i.e., children nested within classrooms within schools), the *type = complex* command was used in Mplus to reduce potential biases in standard errors that could arise as a result of data non-independence.

## Results

### Preliminary Analyses

#### Missing Data Analyses

An examination of missing data and participant attrition revealed that, for all study variables, approximately 18.3% of the data were missing. Missing data on the peer report measures (i.e., aggression and peer acceptance) ranged from 21.1 to 26.8% across time, with rates of missing data being slightly higher by Grade 4. Missing data on teacher reports of prosocial behavior increased from Grade 1 (13.8%) to Grade 4 (32.7%). To assess whether there were any observable or systematic patterns of missing data, a series of univariate *t*-tests were performed to examine the associations among children’s gender, race, ethnicity, and socio-economic adversity on rates of missing data across time. However, there were no clear patterns which indicated that these demographic factors were associated with participant attrition or drop out. Missing data was handled by using full information maximum likelihood (FIML) estimation. This approach is advantageous compared to more traditional missing data techniques because it includes all participants in the analyses regardless of whether they had missing data or dropped out of the study (Enders, 2010).

#### Descriptive Statistics and Bivariate Correlations

Descriptive statistics and bivariate correlations for all study variables are reported in Table 1. The bivariate correlations indicated that aggression, prosocial behavior, and peer acceptance exhibited moderate stability from Grades 1 to 4. Aggression was moderately negatively correlated with prosocial behaviors and peer acceptance. Prosocial behaviors and peer acceptance were moderately positively correlated.

**TABLE 1:** Bivariate correlations and descriptive statistics of study variables.

	1	2	3	4	5	6	7	8	9	10	11	12	13	14	15	16
(1) Aggression (G1)																
(2) Aggression (G2)	0.45**															
(3) Aggression (G3)	0.44**	0.52**														
(4) Aggression (G4)	0.46**	0.43**	0.49**													
(5) Prosocial behavior (G1)	−0.37**	−0.37**	−0.32**	−0.26**												
(6) Prosocial behavior (G2)	−0.30**	−0.39**	−0.35**	−0.33**	0.45**											
(7) Prosocial behavior (G3)	−0.30**	−0.36**	−0.42**	−0.31**	0.35**	0.42**										
(8) Prosocial behavior (G4)	−0.21**	−0.35**	−0.35**	−0.23**	0.36**	0.44**	0.48**									
(9) Peer acceptance (G1)	−0.35**	−0.32**	−0.21**	−0.19**	0.37**	0.30**	0.23**	0.24**								
(10) Peer acceptance (G2)	−0.26**	−0.37**	−0.14**	−0.19**	0.28**	0.31**	0.26**	0.20**	0.46**							
(11) Peer acceptance (G3)	−0.22**	−0.22**	−0.19**	−0.21**	0.25**	0.29**	0.28**	0.28**	0.42**	0.50**						
(12) Peer acceptance (G4)	−0.33**	−0.29**	−0.16**	−0.27**	0.32**	0.28**	0.27**	0.31**	0.44**	0.50**	0.52**					
(13) Gender (boys = 1)	0.28**	0.23**	0.28**	0.32**	−0.19**	−0.24**	−0.22**	−0.27**	–0.04	–0.02	–0.06	−0.10*				
(14) African American	0.18**	0.16**	0.23**	0.20**	−0.17**	−0.20**	−0.21**	−0.15**	−0.15**	−0.16**	−0.11**	−0.08*	–0.02			
(15) Hispanic/Latino	−0.09*	−0.15**	−0.14**	−0.11**	0.12**	0.13**	0.10*	0.11**	0.22**	0.25**	0.31**	0.23**	–0.01	−0.43**		
(16) Socioeconomic adversity	0.09*	0.11**	0.15**	0.12**	−0.12**	−0.13**	–0.08	–0.04	0.00	0.08*	0.15**	0.06	0.00	0.30**	0.24**	
*N*	602	582	619	575	676	621	547	528	601	579	617	574	784	784	784	776
Minimum	–1.24	–1.28	–1.11	–1.14	–2.79	–2.70	–2.71	–2.79	1.50	1.18	1.40	1.00	0.00	0.00	0.00	–1.27
Maximum	4.08	3.75	3.92	4.43	1.17	1.08	1.13	1.14	4.91	4.90	4.83	4.77	1.00	1.00	1.00	1.66
Mean	0.02	0.05	0.10	0.02	0.00	0.00	0.00	0.00	3.41	3.30	3.17	3.10	0.53	0.23	0.37	0.04
*SD*	0.98	1.00	1.04	1.01	1.00	1.00	1.00	1.00	0.69	0.70	0.64	0.64	0.50	0.42	0.48	0.74

G, grade. *p < 0.05; **p < 0.01.

### Developmental Trajectories of Direct Aggression and Prosocial Behaviors

Distinct and heterogeneous trajectory classes were identified from Grades 1 to 4 by first examining aggression and prosocial behaviors independently. These models were used as the basis for determining the optimal number of trajectory classes for each behavioral domain. To determine the optimal number of trajectory classes, multiple fit indices were evaluated in addition to examining whether the trajectory classes appeared substantively and conceptually meaningful (Ram and Grimm, 2009). Fit indices consisted of a combination of multiple information criteria (AIC, BIC, and sample-size adjusted BIC or SABIC), the likelihood ratio test (Lo-Mendell-Rubin likelihood ratio test; LMR-LRT), and classification accuracy. Models with smaller AIC, BIC, and SABIC values indicate better solutions. A significant *p*-value on the LMR-LRT indicates that a model with *k* classes has better fit to the observed data than a model with *k – 1* classes. Classification accuracy was assessed by examining entropy and class assignment probabilities (with values closer to 1 indicating more precise classification).

#### Direct Aggression

The first set of models examined children’s aggression trajectories from Grades 1 to 4. Fit indices for the models with varying classes are reported in Table 2. The 4-class solution had the lowest BIC and SABIC, and the LMR-LRT was statistically significant when comparing the 4-class and 3-class models. Taken together, these fit indices provided support for selecting the 4-class model as the optimal solution. The 4-class model consisted of 15.8% of children with high levels of aggression, 26.1% with moderate aggression, 19.1% with moderate-low aggression, and 39.0% with low aggression. All of the aggression trajectories maintained relatively stable rates of aggression over time.

**TABLE 2:** Fit indices for models examining trajectories of aggressive and prosocial behaviors.

Model	LogL	AIC	BIC	SABIC	Entropy	LMR-LRT	*p*
**Aggression**							
2-Class	−2291.63	4597.26	4629.58	4607.35	0.86	2114.88	***
3-Class	−2133.15	4288.29	4339.08	4304.15	0.77	305.43	**
**4-Class**	**−2097.49**	**4224.98**	**4294.24**	**4246.61**	**0.68**	**68.71**	*
5-Class	−2091.34	4220.68	4308.41	4248.08	0.71	11.85	
6-Class	−2085.51	4217.02	4323.22	4250.19	0.68	9.91	
**Prosocial Behaviors**							
2-Class	−3048.68	6111.35	6143.91	6121.68	0.82	607.26	**
3-Class	−2901.60	5825.19	5876.35	5841.42	0.84	283.50	***
**4-Class**	**−2833.76**	**5697.53**	**5767.28**	**5719.65**	**0.75**	**130.75**	***
5-Class	−2822.33	5682.65	5771.01	5710.67	0.75	22.05	
6-Class	−2814.94	5675.89	5782.84	5709.81	0.74	18.62	

LogL, Loglikelihood; AIC, Akaike information criteria; BIC, Bayesian information criteria; SABIC, sample-size adjusted Bayesian information criteria; LMR-LRT, Lo-Mendell-Rubin likelihood ratio test; G, grade. Class solutions highlighted in bold were selected as the optimal model for each set of analyses. * *p* < 0.05, ** *p* < 0.01, *** *p* < 0.001.

#### Prosocial Behaviors

The second set of models examined children’s prosocial trajectories from Grades 1 to 4. Fit indices for the models with varying classes are reported in Table 2. Although the AIC and SABIC slightly favored models with increasing numbers of classes, the BIC for the 4-class model was the lowest. Moreover, the LMR-LRT was statistically significant when comparing the 4-class to the 3-class model, but not for models with additional classes (i.e., the 5- and 6-class models). Taken together, these fit indices provided support for selecting the 4-class model as the optimal solution. The 4-class model consisted of 5.4% of children with high levels of prosocial behaviors, 15.7% with moderate-high prosocial behaviors, 58.0% with moderate prosocial behaviors, and 21.0% with low prosocial behaviors. All of the prosocial behavior trajectories maintained relatively stable rates over time.

#### Joint Trajectory Models

**FIGURE 1:**
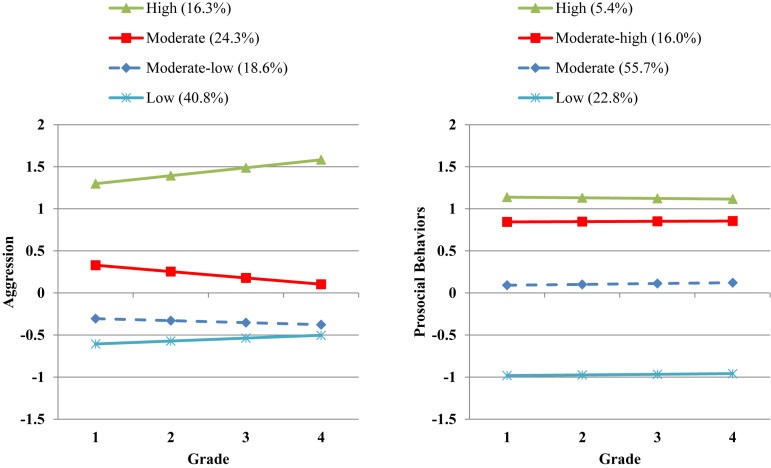
Developmental trajectories (and class percentages) for aggression and prosocial behaviors from Grades 1 to 4.

Based on the results of the growth mixture models in which aggression and prosocial behaviors were examined separately, a 4 × 4 class sequential process growth mixture model was specified by examining aggression and prosocial behaviors simultaneously. More specifically, the trajectory classes identified in this model (see Figure 1) consisted of 16.3% of children with high levels of aggression, 24.3% with moderate aggression, 18.6% with moderate-low aggression, and 40.8% with low aggression. About 5.4% of children had high levels of prosocial behaviors, 16.0% had moderate-high prosocial behaviors, 55.7% had moderate prosocial behaviors, and 22.8% had low prosocial behaviors. Notably, the identified trajectory classes in the joint trajectory model were very similar to the models in which aggression and prosocial behaviors were examined separately; however, there were small changes in the proportions of children in each class.

The joint trajectory model also allowed for an examination of the joint distribution probabilities (i.e., the likelihood that children would exhibit specific combinations of aggression and prosocial behavior). Results indicated a possibility of 16 joint trajectory classes (i.e., 4 aggression trajectory classes in combination with 4 prosocial trajectory classes; see Table 3). The distribution of children across these classes varied considerably. For instance, among the 16.3% of children with high aggression trajectories, the vast majority (87.4%) had low prosocial behavior. However, among the 24.3% of children with moderate aggression trajectories, there was more variability in prosocial behavior such that 65.1% also had moderate prosocial behaviors, and 30.7% had low prosocial behaviors. Among the 21.4% of children with moderate-high or high prosocial trajectories, the vast majority (93.4%) had low or moderate-low aggression trajectories. The largest co-occurring class, consisting of roughly one in four children (24.1%), exhibited a combination of low aggression and moderate prosocial trajectories.

**TABLE 3:** Cross-tabulations examining the frequencies and percentages of children’s co-occurring aggression and prosocial trajectory classes.

	Prosocial trajectories			
Aggression trajectories	High	Moderate-high	Moderate	Low
**Frequencies**				
High	0	3	13_d_	111_a_
Moderate	4	4	123_e_	58_b_
Moderate-low	6_c_	26_c_	110_f_	3
Low	32_c_	92_c_	188_f_	6
**Total percentages**				
High	0.0%	0.4%	1.7%	14.2%
Moderate	0.5%	0.5%	15.8%	7.4%
Moderate-low	0.8%	3.3%	14.1%	0.4%
Low	4.1%	11.8%	24.1%	0.8%
**Row percentages**				
High	0.0%	2.4%	10.2%	87.4%
Moderate	2.1%	2.1%	65.1%	30.7%
Moderate-low	4.1%	17.9%	75.9%	2.1%
Low	10.1%	28.9%	59.1%	1.9%
**Column percentages**				
High	0.0%	2.4%	3.0%	62.4%
Moderate	9.5%	3.2%	28.3%	32.6%
Moderate-low	14.3%	20.8%	25.3%	1.7%
Low	76.2%	73.6%	43.3%	3.4%

*Subscripts shown next to the frequencies represent how the co-occurring trajectory classes were combined to create more parsimonious subgroups: a, high aggression; b, moderate aggression; c, high prosocial; d, high aggression-moderate prosocial; e, moderate aggression-prosocial; f, low aggression-moderate prosocial (reference group). Groups without a subscript were removed.*

### Associations Among Children’s Peer Acceptance Trajectories and Their Co-occurring Aggression and Prosocial Trajectories

The final set of analyses assessed the dynamic and co-occurring longitudinal associations between children’s co-occurring aggression and prosocial trajectory classes with their peer acceptance. Because some co-occurring trajectory classes exhibited very small frequencies, it was not possible to examine every possible combination of the co-occurring aggression and prosocial trajectory classes. Moreover, some trajectory classes were combined in this step of the analysis in order to simplify the presentation of results, provide for a more parsimonious comparison of substantively distinct subgroups, and increase statistical power. This approach resulted in six distinct co-occurring subgroups (these six classes are noted in the subscripts shown in Table 3).

The first group was labeled as the *high aggression group* and consisted of 14.2% of children (*n* = 111) who exhibited high aggression trajectories in combination with low prosocial trajectories. The second group was labeled as the *moderate aggression group* and consisted of 7.4% of children (*n* = 58) who exhibited moderate aggression trajectories in combination with low prosocial trajectories. The third group was labeled as the *high prosocial group* and consisted of 20.0% of children (*n* = 156) with either high or moderate-high prosocial trajectories in combination with low or moderate-low aggression trajectories. The fourth group was labeled as the *high aggression-moderate prosocial group* and consisted of 1.7% of children (*n* = 13) with high aggression trajectories in combination with moderate prosocial trajectories. The fifth group was labeled as the *moderate aggression-prosocial* group and consisted of 15.8% of children (*n* = 123) with moderate aggression trajectories in combination with moderate prosocial trajectories. The sixth and final group was labeled as the *moderate prosocial group* and consisted of 38.3% of children (*n* = 298) with low or moderate-low aggression trajectories in combination with moderate prosocial trajectories. About 2.6% of children (*n* = 20) from six subgroups were removed during this step of the analysis because they were in groups with very small frequencies, ranging from 0 to 6 children, and we did not have a logical rationale for combining them with other groups (these subgroups do not contain a subscript in Table 3).

To examine the development of children’s peer acceptance from Grades 1 to 4, conditional latent growth models were specified. In these growth models, the six group assignments for the co-occurring aggression and prosocial trajectory classes were used to compute a series of dummy-coded grouping variables that were then regressed on the latent intercept and slope factors (using the moderate prosocial group as the referent). Models were specified two times by adjusting the intercept to estimate group differences in children’s peer acceptance in Grades 1 and 4. Notably, adjusting the intercept in these models did not change model fit or the form of the trajectory that was estimated. Fit for all growth models was deemed adequate if RMSEA < 0.06, and SRMR < 0.08 (Hu and Bentler, 1999). Because software packages may use an inappropriate baseline model to compute the CFI in growth curve models (Wu et al., 2009), this index was not interpreted for these models. In addition to examining the effects of children’s aggression and prosocial trajectories, these models also controlled for gender, ethnicity, race, and socioeconomic adversity.

**FIGURE 2:**
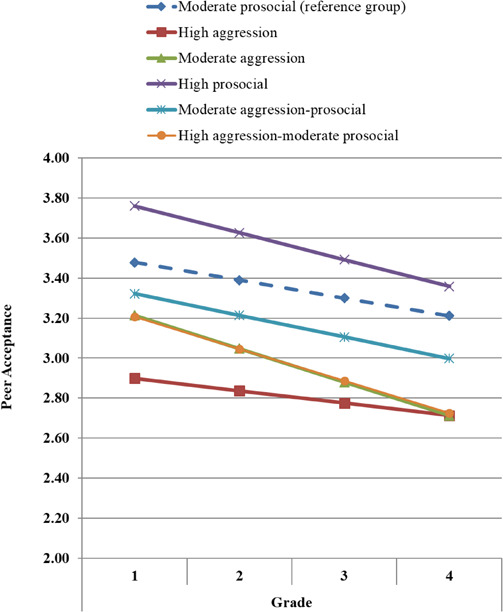
Children’s predicted peer acceptance trajectories by co-occurring aggression and prosocial trajectory classes.

Estimates and significance tests for the conditional growth model (*χ^2^* = 26.94, *df* = 23, *p* = 0.26; RMSEA = 0.02; SRMR = 0.02) are reported in Table 4, and illustrated for interpretative purposes in Figure 2. Compared to the reference group (i.e., the moderate prosocial group), the high aggression group had significantly lower peer acceptance in Grade 1, which was sustained through Grade 4. The moderate aggression group had significantly lower peer acceptance in Grades 1 and 4, and a significant declining slope in peer acceptance from Grades 1 to 4. The high prosocial group had significantly higher peer acceptance in Grades 1 and 4; however, this group exhibited a significant decline in peer acceptance from Grades 1 to 4. The moderate aggression-prosocial group had significantly lower peer acceptance in Grades 1 and 4. Finally, the high aggression-moderate prosocial group had significantly lower peer acceptance in Grade 4, but not in Grade 1. In summary, all of the groups were significantly different from the reference group in both Grades 1 and 4, with the exception of the high aggression-moderate prosocial group in Grade 1. Compared to the reference group, the high prosocial group had significantly higher levels of peer acceptance, and all other groups had lower levels of peer acceptance across time.

**TABLE 4:** Estimates for conditional growth models examining children’s peer acceptance.

	Peer Acceptance		
Effects	*Est*	*SE*	*p*
**Grade 1**			
High aggression	−0.58	0.08	***
Moderate aggression	−0.26	0.08	***
High prosocial	0.28	0.06	***
Moderate aggression-prosocial	−0.16	0.06	*
High aggression-moderate prosocial	−0.27	0.30	
Gender (boys)	0.12	0.05	*
African American	−0.06	0.07	
Hispanic	0.24	0.06	***
Socioeconomic Adversity	0.05	0.04	
**Grade 4**			
High aggression	−0.50	0.07	***
Moderate aggression	−0.50	0.08	***
High prosocial	0.15	0.05	**
Moderate aggression-prosocial	−0.21	0.07	**
High aggression-moderate prosocial	−0.49	0.24	*
Gender (boys)	0.07	0.05	
African American	0.17	0.06	**
Hispanic	0.33	0.07	***
Socioeconomic Adversity	0.07	0.03	*
**G1–G4 slope**			
High aggression	0.03	0.03	
Moderate aggression	−0.08	0.04	*
High prosocial	−0.05	0.02	*
Moderate aggression-prosocial	−0.02	0.03	
High aggression-moderate prosocial	−0.07	0.13	
Gender (boys)	−0.02	0.02	
African American	0.08	0.02	***
Hispanic	0.03	0.02	
Socioeconomic Adversity	0.01	0.01	

G, grade. The moderate prosocial group was used as the reference group. *p < 0.05; **p < 0.01; ***p < 0.001.

Additional analyses more explicitly evaluated the hypothesis that prosocial behaviors buffered the effects of aggression on peer acceptance. By changing the reference group, the moderate aggression group was directly compared with the moderate aggression-prosocial group in order to assess whether comparable levels of aggression in combination with varying levels of prosocial behavior were differentially associated with peer acceptance. Results indicated that the moderate aggression group had significantly lower peer acceptance in Grade 4 (*b* = −0.29, *p* < 0.01), but not in Grade 1 (*b* = −0.11, *p* = 0.18). Similar analyses were performed to compare the high aggression-moderate prosocial group with high aggression group, however these effects were not statistically significant (in Grade 1: *b* = 0.31, *p* = 0.28; in Grade 4: *b* = 0.01, *p* = 0.95).

To evaluate the hypothesis that moderate aggression was more adaptive than high aggression, additional group comparisons were made. First, the results indicated that the moderate aggression group had significantly higher peer acceptance than the high aggression group in Grade 1, but not in Grade 4 (*b* = 0.32, *p* < 0.001 and *b* = 0.00, *p* = 1.00, respectively). Second, the differences between the high aggression-moderate prosocial group and the moderate aggression-prosocial group were not statistically significant (in Grade 1: *b* = −0.12, *p* = 0.70; in Grade 4: *b* = −0.27, *p* = 0.24).

These models also controlled for gender, ethnicity, race, and socioeconomic status. Results indicated that boys had significantly higher peer acceptance in Grade 1, but not in Grade 4. African American children had significantly higher peer acceptance in Grade 4, and a significant increasing slope in peer acceptance from Grades 1 to 4. Hispanic children had significantly higher peer acceptance in Grades 1 and 4. Socioeconomic adversity was associated with higher rates of peer acceptance in Grade 4.

As previously indicated, several groups identified in the joint trajectory analyses were combined to create more parsimonious groups, increase statistical power, and to facilitate the group comparisons. Although a logical approach was used to combine groups that were qualitatively similar (i.e., moderate-high and high prosocial behaviors; moderate-low and low aggression), it was possible that this process could have unintentionally combined groups with distinct peer acceptance trajectories. Consequently, a model was estimated to validate the process of combining different subgroups. More specifically, a conditional growth model was estimated in which peer acceptance trajectories were examined separately for each of the joint trajectory classes (i.e., without combining any subgroups). That is, rather than aggregate the four classes consisting of low or moderate-low aggression co-occurring with high or moderate-high prosocial behaviors into a high prosocial group (see Table 3), each of these classes was examined separately. Notably, because one of these classes was relatively small (n = 6), it was removed from this part of the analyses. Results indicated that children in the remaining three groups were quite similar to one another (*M*_*intercept*_ = 3.69–3.98 and *M*_*slope*_ = −0.13 to −0.15; in comparison to *M*_*intercept*_ = 3.76 and *M*_*slope*_ = −0.13 for the combined group), and exhibited higher rates of peer acceptance across time compared to children in other groups. Moreover, the two groups combined to create the moderate prosocial group (*M*_*intercept*_ = 3.37–3.55 and *M*_*slope*_ = −0.07 to −0.10; in comparison to *M*_*intercept*_ = 3.48 and *M*_*slope*_ = −0.09 for the combined group) exhibited trajectories of peer acceptance that were lower than the (three) high prosocial classes, but higher than the (four) aggressive classes.

## Discussion

The findings of this study contribute to our understanding of the co-occurring or joint development of children’s direct aggression and prosocial behaviors in middle childhood (Grades 1 to 4). The results revealed several distinct trajectory profiles which differentiated children who were highly aggressive *or* highly prosocial from those who exhibited a combination of aggressive and prosocial behavioral styles. The results also elucidated how children’s prosocial behaviors buffered the effects of direct aggression on children’s peer acceptance, such that moderately aggressive-prosocial children exhibited higher peer acceptance, by Grade 4, compared to children with similar levels of aggression who were not prosocial. However, moderate aggression, with or without co-occurring prosocial behaviors, was associated with lower peer acceptance compared to low aggression.

### Co-occurring (Joint) Development of Direct Aggression and Prosocial Behaviors

The first step in the analyses was to examine each behavioral style independently. With respect to children’s aggression trajectories, the model fit indices indicated that the 4-class model was the optimal solution. Although we hypothesized identifying three classes, the trajectory classes identified in the 4-class model were mostly consistent with expectations. Three of the classes consisted of children with relatively stable low, moderate, and high aggression trajectories. In addition to these classes, a fourth class emerged that consisted of children with moderate-low aggression; however, this class exhibited a trajectory pattern that was quite similar to the low aggression class. Taken together, although roughly 60% of children had either low or moderate-low aggression, a small but substantial proportion of children (16.3%) exhibited chronic aggression and were persistently one standard deviation above the mean with respect to their aggression trajectories across Grades 1 to 4. These findings corroborate other studies which have consistently identified a subgroup of chronically aggressive children (Broidy et al., 2003; Ettekal and Ladd, 2009).

The model fit indices for children’s prosocial behavior trajectories also indicated that the 4-class model was the optimal solution. For the most part, the trajectory classes identified in this model were consistent with expectations. As hypothesized, three of the classes were characterized by relatively stable low, moderate, and high prosocial behaviors. In addition, a fourth class emerged that consisted of children with moderate-high prosocial behavior; however, this class exhibited a trajectory pattern that was very similar to the high prosocial class. As hypothesized, the majority of children (about 56%) exhibited moderate prosocial behaviors; however, a substantial percentage of children were characterized by either very low levels of prosocial behaviors (22.8%), roughly one standard deviation below the mean, or relatively high prosocial behaviors (21.4%) which were about one standard deviation above the mean. Taken together, these findings are consistent with other studies which have used similar methods to examine children’s prosocial trajectories (Côté et al., 2002; Kokko et al., 2006; Nantel-Vivier et al., 2009, 2014; Flynn et al., 2015).

Although there have been a substantial number of studies which have examined children’s aggressive and prosocial behaviors separately, it has been less common for researchers to investigate their joint development. Toward this end, this study provided insights into the co-occurring development of direct aggression and prosocial behaviors as children progressed through elementary school. Because some co-occurring classes were highly unlikely, and after combining classes which appeared to be similar, we evaluated six distinct co-occurring subgroups.

Two of these classes, consisting of about 22% of participating children, were primarily characterized by their higher levels of direct aggression and low levels of prosocial behaviors (i.e., moderate and high aggressors). The identification of these two classes is consistent with the premise that many aggressive children are unlikely to utilize prosocial behaviors. However, this characterization appears to be particularly applicable to children who were highly aggressive. That is, about 87% of children identified as being highly aggressive had low prosocial behaviors. This finding provides support for social cognitive deficit perspectives of aggression, according to which chronically aggressive children are likely to lack social skills and self-regulatory competencies that would facilitate the use of non-aggressive behavioral styles (Ettekal and Ladd, 2015a; Olson et al., 2017). For instance, high (chronic) aggressors are likely to exhibit lower effortful control and theory of mind (Olson et al., 2017), which may hinder their ability to more effectively engage in prosocial behaviors with peers.

In contrast to high aggressors, children who engaged in moderate aggression exhibited substantially greater variability with respect to their co-occurring prosocial behaviors. For instance, among children who exhibited moderate aggression trajectories, about 31% had low prosocial behaviors in comparison to about 65% who had co-occurring moderate prosocial behaviors. Taken together, these findings imply that although some moderate aggressors may exhibit social-cognitive biases and dispositions similar to high aggressors, there appears to be considerable heterogeneity among children who engage in moderate uses of aggression.

Among children who were either moderately or highly aggressive, we differentiated those with co-occurring moderate prosocial behaviors from those with low prosocial behaviors. Notably, we did not reliably identify subgroups of (moderately or highly) aggressive children who had high prosocial trajectories. These groups appeared to be fairly consistent with previous studies that have used a similar methodology. Jambon et al. (2019) did not identify a subgroup with high aggression and prosocial behaviors; however, they identified two subgroups (which they labeled as desisting and escalating) who exhibited moderate levels of co-occurring aggression and prosocial behaviors. Kokko et al. (2006) identified a subgroup of highly aggressive boys with moderate prosocial behaviors (but did not identify a high prosocial trajectory class). Nantel-Vivier et al. (2014) found that among younger children with moderate and high aggression trajectories, it was more common to have moderate as opposed to high prosocial behaviors. Taken together, our findings and those of other investigators imply that there is considerable heterogeneity in the behavioral styles of children who act aggressively, such that some are more capable of balancing their use of direct aggression with prosocial behaviors. Moreover, children who engage in both behavioral styles exhibit a pattern in which they use these behaviors in moderation compared to peers (Ciarrochi et al., 2019; Jambon et al., 2019; Hartl et al., 2020).

In addition to these subgroups of children who engaged in direct aggression, there were also a substantial number of children who were characterized as being primarily prosocial. More specifically, because the low and moderate-low aggression trajectory classes exhibited a similar developmental pattern, as did the high and moderate-high prosocial trajectories, these classes were combined for parsimony, and labeled as the high prosocial group, consisting of about 20% of children. An additional 38% of children were also identified with moderate prosocial trajectories (in combination with low and moderate-low aggression). Taken together, these findings revealed that the majority of children (roughly 3 in 5) abstained from using direct aggression and were primarily characterized by their prosocial behavioral styles.

### Associations Among Children’s Peer Acceptance Trajectories and Their Co-occurring Aggression and Prosocial Trajectories

#### Is Prosocial Behavior a Protective Factor for Aggressive Children?

Our second aim was to chart the development (continuity and changes) in peer acceptance (from Grades 1 to 4) for each of the co-occurring subgroups identified in Aim 1. We hypothesized a protective effect of prosocial behaviors among aggressive children such that those who engaged in co-occurring aggressive and prosocial behaviors were more likely to have higher levels of peer acceptance as they transitioned from middle to late childhood, compared to children who were primarily (only) aggressive. The rationale for this hypothesis was based on the proposition that children who engage in co-occurring aggressive and prosocial behaviors are able to pursue two salient social goals, namely maintaining power (‘getting ahead’) and social harmony (‘getting along’; see Hogan, 1982). That is, although their aggressive behaviors may have antagonized some of their peers, these children are able to more effectively balance their use of aggression with prosocial behaviors to reduce potential social sanctions and retributions, thus ameliorating the potentially negative effects of aggression (Roseth et al., 2011).

Two specific group comparisons were considered to evaluate this hypothesis. First, we found that the moderate aggression-prosocial group had significantly higher peer acceptance in Grade 4 compared to the moderate aggression group. The differences between these two groups became more pronounced over time as the moderate aggression group exhibited a steeper decline in peer acceptance from Grades 1 to 4. These findings imply that among moderate aggressors, the use of moderate prosocial behaviors may provide some benefits with respect to maintaining their peer acceptance. However, it is important to note that although the moderate aggression-prosocial group fared well when compared to moderate aggressors, their levels of peer acceptance were significantly lower than children who engaged in low aggression and moderate prosocial behaviors (i.e., the reference group). Thus, prosocial behaviors may exert some protective functions; however, the use of direct aggression, even at a moderate level and co-occurring with prosocial behaviors, continues to be a risk factor for lower peer acceptance.

Second, we compared the peer acceptance trajectories of the high aggression versus high aggression-moderate prosocial groups. Although the observed differences were in the expected direction in Grade 1, they were not statistically significant (at *p* < 0.05). It is plausible that when children engage in high and chronic levels of direct aggression, their co-occurring use of prosocial behaviors are less effective. That is, these children may develop a reputation as being excessively and persistently aggressive, and even when they try to engage in some prosocial behaviors, these attempts at amelioration are not as well received by their peers, who continue to dislike them. This interpretation is consistent with the findings which revealed that the peer acceptance trajectories for these two groups converged (i.e., became more similar) over time.

#### Is Moderate Aggression Adaptive?

We also hypothesized that moderate (as opposed to high) levels of aggression would be more adaptive with respect to children’s peer acceptance. We considered several group comparisons to evaluate this hypothesis, and taken together, the findings did not consistently corroborate this hypothesis. The first comparison evaluated children with different levels of aggression (i.e., moderate versus high) in combination with low prosocial behaviors. The moderate aggression group had significantly higher peer acceptance than the high aggression group in Grade 1 only and exhibited a significant decline in peer acceptance over time, which resulted in similar levels of peer acceptance by Grade 4. Taken together, these results imply that some (moderate) use of aggression may be more normative among younger children (i.e., at school entry), but become less socially acceptable as children mature and develop more advanced self-regulation and social-cognitive skills (e.g., perspective-taking, conflict resolution). Consequently, for children who persist in using moderate levels of direct aggression, in the absence of other behavioral styles (e.g., prosocial behavior), there may be greater declines in peer acceptance over time.

The second comparison evaluated children with different levels of aggression in combination with moderate prosocial behaviors (i.e., moderate aggression-prosocial group and high aggression-moderate prosocial group). Although the differences in peer acceptance between these two groups were in the expected direction, and became more pronounced over time, they were not statistically significant. It is possible that this analysis lacked sufficient statistical power to detect a meaningful difference among these subgroups due to the small sample size of the high aggression-moderate prosocial group.

### Limitations, Future Directions, and Implications

This study’s findings should be evaluated in consideration of its potential limitations. In assessing each of these limitations, we also highlight how future research may advance the findings reported in the current study. Perhaps most importantly, there are several methodological and measurement related considerations that warrant further attention. With respect to this study’s focus on direct aggression, one potential direction for future research may be to compare direct versus indirect forms of aggression (e.g., relational and social aggression). For instance, relational aggression may be used in more functional ways to advance children’s social positions and reputations (Ettekal and Ladd, 2015a). For similar reasons, it may also be important to distinguish instrumental or proactive functions of aggression (e.g., bullying) from reactive aggression. With respect to the measurement of prosocial behaviors, researchers have increasingly conceptualized its multi-dimensional nature (Eisenberg and Fabes, 1998; Carlo et al., 2003). Consequently, an interesting direction for future research is to more explicitly distinguish how its multiple forms are associated with children’s peer acceptance.

There are also some methodological considerations pertaining to measurement timing and informant type. With respect to timing, although the findings indicated that children’s developmental trajectories across multiple years appear to be relatively stable, there is evidence that children may exhibit greater fluctuations in their behavioral styles across shorter time intervals. For instance, Roseth et al. (2011) found that within one school year, children’s coercive behaviors were initially higher at that start of the school year, presumably to establish their social positions, but subsequently declined. In contrast, prosocial behaviors increased during the school year, as children used these behaviors to maintain their social positions. An interesting direction for future research would be to examine children’s developmental trajectories using a research design that is more sensitive at capturing both short-term and long-term variations (e.g., collecting assessments across several years with multiple assessments collected within each school year). In addition to assessment timing, it may be important to use multi-informant data which are less reputation based than peer reports. That is, because peer reports often rely on a child’s reputation within their peer group, they may not be as sensitive at capturing short-term fluctuations in children’s behavioral styles compared to other methods (e.g., observational measures).

This study focused on one specific aspect of children’s relational development (i.e., peer group acceptance). Considering that peer acceptance is viewed as a measure of children’s sociometric popularity, and is measured within a child’s collective peer group (e.g., classmates), one direction for future research may be to investigate the impact of children’s behavioral styles at the dyadic (rather than peer group) level by assessing how these behaviors are associated with their friendships and friendship qualities. Furthermore, it may also be important to differentiate same-sex and mixed friendships (see Ciarrochi et al., 2019). Several investigators have also evaluated how there may be differential effects of aggression on children’s sociometric versus perceived popularity (Cillessen and Mayeux, 2004; Cillessen and Rose, 2005). However, because there was not a measure of perceived popularity available in the current study, it was not possible to differentiate these two constructs. Although these two constructs tend to be moderately and positively correlated, they have distinct etiologies. Thus, it would have been interesting to examine whether the trajectory groups identified in this study would have exhibited distinct developmental patterns with respect to peer acceptance compared to perceived popularity (Hawley, 2003a; Hartl et al., 2020).

Finally, the correlational and analytic design limited causal inferences. Because the overarching aims of this study were to examine the co-occurring development of direct aggression, prosocial behaviors, and peer acceptance, it was outside the scope of this study to assess the direction of effect or causality among these constructs. The use of variable centered models, and more specifically, variants of full panel cross-lagged models, may help to further disentangle the direction of effect among these constructs (e.g., Zimmer-Gembeck et al., 2005; Obsuth et al., 2015).

Although the primary aims of this study were to advance our understanding of how children’s co-occurring aggressive and prosocial behavioral styles and peer acceptance develop in middle childhood, these findings may also have implications for intervention efforts that aim to reduce children’s school-based aggression. By considering the potential heterogeneity among aggressive children, intervention programs may gain insights pertaining to their program effects, and the extent to which a program may have varying impacts on different children. For instance, it is possible that social skills training programs may be more effective for aggressive children with low prosocial behaviors; however, these efforts may be less efficacious when aggressive children are capable of being prosocial, but nonetheless resort to using aggression. For these children, intervention efforts may need to provide a more holistic approach that targets other developmental domains (e.g., empathy training) as well as contextual processes (e.g., social norms). Perhaps for these reasons, bystander interventions which target pro-aggression social norms appear to be particularly effective in hindering the use of aggression to attain social resources (Polanin et al., 2012).

## Data Availability Statement

The datasets presented in this study can be found in online repositories. The names of the repository/repositories and accession number(s) can be found below: Data from this project are publicly available for secondary data analysis at the National Institute of Child Health and Human Development (NICHD) Data and Specimen Hub (DASH; https://dash.nichd.nih.gov/study/14412).

## Ethics Statement

The studies involving human participants were reviewed and approved by Texas A&M University (Human Research Protection Program). Written informed consent to participate in this study was provided by the participants’ legal guardian/next of kin.

## Author Contributions

IE: conceptualization, formal analysis, methodology, visualization, and writing (original draft preparation, review, and editing). MM: literature review and writing (original draft preparation, review, and editing). Both authors contributed to the article and approved the submitted version.

## Conflict of Interest

The authors declare that the research was conducted in the absence of any commercial or financial relationships that could be construed as a potential conflict of interest.

## Footnotes

1 https://dash.nichd.nih.gov/study/14412
